# Drought Response in Wheat: Key Genes and Regulatory Mechanisms Controlling Root System Architecture and Transpiration Efficiency

**DOI:** 10.3389/fchem.2017.00106

**Published:** 2017-12-05

**Authors:** Manoj Kulkarni, Raju Soolanayakanahally, Satoshi Ogawa, Yusaku Uga, Michael G. Selvaraj, Sateesh Kagale

**Affiliations:** ^1^Canadian Wheat Improvement Flagship Program, National Research Council Canada (NRC-CNRC), Saskatoon, SK, Canada; ^2^Saskatoon Research and Development Centre, Agriculture and Agri-Food Canada, Saskatoon, SK, Canada; ^3^Department of Global Agricultural Sciences, Graduate School of Agricultural and Life Sciences, The University of Tokyo, Tokyo, Japan; ^4^Institute of Crop Science (NICS), National Agriculture and Food Research Organization (NARO), Tsukuba, Japan; ^5^Agrobioversity Research Area, International Center for Tropical Agriculture (CIAT), Cali, Colombia

**Keywords:** wheat, drought, root traits, transpiration efficiency, transcriptional regulation, EAR motif

## Abstract

Abiotic stresses such as, drought, heat, salinity, and flooding threaten global food security. Crop genetic improvement with increased resilience to abiotic stresses is a critical component of crop breeding strategies. Wheat is an important cereal crop and a staple food source globally. Enhanced drought tolerance in wheat is critical for sustainable food production and global food security. Recent advances in drought tolerance research have uncovered many key genes and transcription regulators governing morpho-physiological traits. Genes controlling root architecture and stomatal development play an important role in soil moisture extraction and its retention, and therefore have been targets of molecular breeding strategies for improving drought tolerance. In this systematic review, we have summarized evidence of beneficial contributions of root and stomatal traits to plant adaptation to drought stress. Specifically, we discuss a few key genes such as, *DRO1* in rice and *ERECTA* in Arabidopsis and rice that were identified to be the enhancers of drought tolerance via regulation of root traits and transpiration efficiency. Additionally, we highlight several transcription factor families, such as, ERF (ethylene response factors), DREB (dehydration responsive element binding), ZFP (zinc finger proteins), WRKY, and MYB that were identified to be both positive and negative regulators of drought responses in wheat, rice, maize, and/or Arabidopsis. The overall aim of this review is to provide an overview of candidate genes that have been identified as regulators of drought response in plants. The lack of a reference genome sequence for wheat and non-transgenic approaches for manipulation of gene functions in wheat in the past had impeded high-resolution interrogation of functional elements, including genes and QTLs, and their application in cultivar improvement. The recent developments in wheat genomics and reverse genetics, including the availability of a gold-standard reference genome sequence and advent of genome editing technologies, are expected to aid in deciphering of the functional roles of genes and regulatory networks underlying adaptive phenological traits, and utilizing the outcomes of such studies in developing drought tolerant cultivars.

## Introduction

Wheat is one of the important staple food crops supplying 20% of calories globally (Lobell and Gourdji, 2012; Shiferaw et al., 2013). Currently, two major wheat species, hexaploid bread wheat (*Triticum aestivum*; 2n = 6x = 42) and tetraploid durum wheat (*Triticum durum*; 2n = 4x = 28), are commercially important. Food and Agriculture Organization (FAO) of the United Nations has estimated 739.9 million tons of wheat production in 2017 (http://www.fao.org/worldfoodsituation/csdb/en/). Global wheat yields have increased at a mere 1.0% per year in the past two decades (Manes et al., 2012). Wheat crop is sensitive to heat and drought stresses mainly at the flowering and grain development stages, which negatively impact the yield and grain quality (lower 1,000 grain weight and change in protein quality). Annual production variability estimated at ~40% was mainly due to heat waves and drought situations in major wheat producing belts throughout the world (Zampieri et al., 2017). Demand for wheat is estimated to increase by 60% by 2050, but production might go down by 29% as a result of climate change imposed environmental stresses (Manickavelu et al., 2012). These predictions indicate that improving abiotic stress tolerance in wheat is paramount for global food security in the near future. Continued wheat genetic improvement is thus critically important as it has direct impact on economic development, food security, and international grain trade.

Most of the stress tolerance traits in wheat are polygenic and hence complex to understand at the physiological and molecular levels. Approaches like quantitative trait locus (QTL) mapping, marker assisted breeding, and introgression from wild gene pool are being employed to improve drought tolerance (Mwadzingeni et al., 2016). A summary of recent efforts in QTL and association mapping for drought tolerance associated traits is provided in Table 1. Success in QTL and association mapping approaches in wheat has unraveled value of these methods as a starting point for fine mapping and identification of genes affecting drought tolerance (Gupta et al., 2017). The recent advances in high-throughput genotyping and phenotyping methods are enabling more advanced approaches such as, genomic selection that allow analysis of the architecture of complex traits.

**Table 1:** QTL and association mapping of drought tolerance traits in wheat.

**Drought tolerance traits**	**Mapping approach**	**Chromosomal location of QTLs**	**Wheat type**	**Stress condition**	**References**
Root development	QTL mapping	7AS	Emmer	Drought	Merchuk-Ovnat et al., 2017
Days to anthesis, grain filling period, 1,000 kernel weight (TKW)	QTL mapping	5A, 7A	Bread	Rainfed condition	Gahlaut et al., 2017
Seeds per spike, number of spikes per plant, TKW, grain yield	QTL mapping	3A, 1A, 7A	Bread	Drought	Xu et al., 2017
Plant Height, days to heading, spike length, seeds per spike, number of spikes per plant	Association mapping	5A, 5B, 6B, 2D, 2B, 6B, 7A, 1B, 4B	Bread	Drought	Mwadzingeni et al., 2017
Photosynthesis, TKW, grain yield	Association mapping	5D, 6D, 7D	Bread	Drought	Saeed et al., 2017
Early ground cover	QTL mapping	6A	Bread	Rainfed condition	Mondal et al., 2017
Plant Height, days to heading, spike length, TKW, grain yield	Association mapping	1B, 2B, 3B, 4B,5B,6B, 7B	Durum		Soriano et al., 2017
Root traits	Association mapping	2B, 5B, 7B, 6D	Bread	Not applicable	Ahmad et al., 2017
Cell wall bound phenolics	QTL mapping	4B, 6R	Triticale	Drought	Hura et al., 2017
Root length	QTL mapping	1BL, 2DS, 5AL, 6AL, 7BL, 3AL	Synthetic hexaploid/Spring wheat/	Water stress	Ayalew et al., 2017
Root and shoot traits	QTL mapping	4B	Durum/*T.dicoccum*	Not applicable	Iannucci et al., 2017
Yield, root morphology	Association mapping	1A, 1B, 4B, 6B	Durum	PEG stress	Lucas et al., 2017
Leaf water content, leaf dry weight, chlorophyll fluorescence	QTL mapping	1,2,3	*Brachypodium distachyon*	Drought	Jiang et al., 2017
Stem water soluble carbohydrates	QTL mapping	4A, 2D	Bread	Drought stress	Nadia et al., 2017
Water soluble carbohydrates	Association mapping	1A, 1B,1D, 4A	Bread	Rainfed	Ovenden et al., 2017
Seedling root traits	QTL mapping	4B, 7A, 7B	Tibetan semi-dwarf wheat	Hydroponics	Ma et al., 2017b

In the recent past, progress has been made in identifying key regulators of drought tolerance in wheat using transgenic approaches. Microarray and RNA sequencing-based gene expression analyses have also been used as important tools in the past to understand wheat responses to varoius abiotic stresses including drought stress. A recent field study looking at the effect of drought on wheat transcriptome changes during reproductive stages detected over 300 differentially expressed genes involved in many critical processes including floral development, photosynthetic activity, and stomatal movement (Ma et al., 2017a). A common response to drought stress involves differential expression of cytochrome P450, heat shock proteins, dehydrins, glutathione transferase, proteinase inhibitors, and regulatory proteins including transcription factors. Several transcription factors, such as, bHLH, bZIP, ERF, HD-ZIP, NAC, and WRKY were differentially expressed in a drought tolerant wheat genotype compared to susceptible genotype (Ergen et al., 2009). Transcriptomic and proteomic analyses of a pale green durum wheat mutant under drought stress indicated expression modulation of several genes encoding antioxidant enzymes, photosystem components, and enzymes representing carbohydrate metabolism and the tricarboxylic acid cycle that may be valuable in addressing drought resistance in wheat (Peremarti et al., 2014). Similarly, a number of other transcriptome and proteome profiling, and genetic manipulation studies have identified candidate genes with potential roles in drought tolerance mechanisms. A summary of promising candidates identified through such studies is provided in Table 2.

**Table 2:** Identification of candidate genes for drought tolerance through transcriptome and proteome profiling, and genetic manipulation.

**Transcriptome or proteome profiling or genetic manipulation studies**	**Differential expression/regulation of genes, pathways**	**Phenotypes**	**References**
Silicon application for drought tolerance enhancement in wheat	Upregulation of antioxidant, ascorbate—glutathione and phenylpropanoid pathway genes	Elevated drought tolerance due to increased chlorophyll content and lower H_2_O_2_, ascorbate and glutathione	Ma et al., 2016
Succinate dehydrogenase inhibitor (SHI) fungicide spray under drought stress	Cell wall expansion, wax, and defense genes	Enhanced drought tolerance	Ajigboye et al., 2017
Overexpression of the wheat expansin gene *TaEXPA2* for improved drought tolerance	Overexpression in tobacco	Enhanced drought tolerance, increased seed production under drought stress in tobacco	Chen et al., 2016
Dehydration and rehydration proteomic analysis	Induction of pathways related to carbohydrate and amino acid metabolism, antioxidants and defense, and ATP synthesis	Drought tolerance	Cheng et al., 2016
Overexpression of *TaWRKY1*	Overexpression in tobacco	Slower water loss from leaves, higher biomass accumulation, enhanced osmolyte, and antioxidant accumulation leading to drought tolerance in tobacco	Ding et al., 2016
Pre-treatment of wheat seedlings with NaHS (sodium hydrosulphide) under drought	SOD, transport, CDPK, ABA, Auxin, ribosome biogenesis	Improved drought tolerance in wheat seedlings	Li et al., 2017
Durum wheat micro-RNA targets	Target genes of micro-RNAs under drought stress: ARFs, HD-Zip, SOD, ROS, HSPs	Modulated drought response	Liu H. et al., 2017
Drought response genes in developing wheat glumes	Enhanced expression of phenylpropanoid biosynthesis pathway genes in wheat glumes	Enhanced drought tolerance	Liu C. et al., 2017
Splice variation in wheat as an effect of drought	*HSFA1FD, HSFA6B*, Heat Shock Protein DnaJ alternatively spliced	Drought tolerance	Liu Z. et al., 2017
Wheat transcriptome changes under drought stress	*LTPL38 and alpha-Amylase3* genes	Enhanced drought tolerance at reproductive phases	Ma et al., 2017a
Response of He-Ne laser pretreated wheat seedlings to drought stress	Altered expression of genes related to photosynthesis, nutrient uptake, and transport	Enhanced drought tolerance in wheat	Qiu et al., 2017
*Aegilops longissima* substitution lines in Chinese spring	Increased expression of ascorbate peroxidase, serpin-Z2B, and alpha amylase genes under drought stress	Drought tolerance trait introduced from wild resources	Zhou et al., 2016

Wheat cultivars have adapted various drought tolerance mechanisms, which include formation of deeper roots, accumulation of higher biomass, exertion of better stomatal control over transpiration (Chipilski et al., 2012), enhancement of osmoprotective and antioxidant response (Huseynova, 2012; Loutfy et al., 2012), and importantly a better coordination of positive and negative regulation of gene expression.

Developmental response of plants to drought stress is manifested through enhanced root growth and suppressed shoot growth resulting in increased root: shoot ratio (Sharp et al., 2004; Yamaguchi and Sharp, 2010; Xu et al., 2013). A combination of 20% faster root descent and more efficient roots can result in more effective water extraction from sub-soil (roots below 60 cm) and provide yield benefits of 0.32–0.44 t/ha in wheat (Lilley and Kirkegaard, 2011). In addition, Rauf et al. (2007) observed up to 50% increase in wheat root: shoot ratio in response to drought stress. Elevated abscisic acid (ABA) levels is shown to function as a promoter of root growth and simultaneously a repressor of shoot growth (Sharp et al., 2004; Xu et al., 2013). Crop's ability to extract water from larger soil volume is critical for yield stability under depleting soil moisture in rainfed production systems. Thus, deep root systems contribute to greater yield potential under drought conditions (Reynolds et al., 2009; Pask and Reynolds, 2013). *DEEPER ROOTING 1* (*DRO1*) gene in rice and related genes in Arabidopsis and *Prunus* species, which will be discussed in detail in the following sections, have been shown to alter root architecture for drought avoidance and improved use of water resources (Uga et al., 2013; Guseman et al., 2017). In addition to improved root traits, greater crop transpiration efficiency (TE) is also critical for yield protection in the agro-ecological regions with limited soil moisture availability (Condon et al., 2004).

This review highlights the progress made in physiological and molecular traits in important crops to maintain yield stability under depleting soil moisture conditions. Here, we focus only on a set of important genes (*DRO1, ERECTA*) and transcription regulators (AP2/ERF, ZFPs, WRKY, and MYB) that are functionally characterized for their role in drought tolerance (Tables 3, 4).

**Table 3:** Examples of transcriptional activators involved in modulation of drought response.

**Gene**	**Identified in plant species**	**Functional validation**	**Phenotype**	**References**
*DEEPER ROOTING* (*DRO1*)	Rice	Overexpression in Arabidopsis, *Prunus* species	Deeper roots	Uga et al., 2011, 2013; Guseman et al., 2017
*MORE ROOT*	Wheat	Overexpression in rice and Arabidopsis	More crown roots in rice and more lateral roots in Arabidopsis	Li et al., 2016
*TaER1 and TaER2*	Wheat	Expression pattern in wheat flag leaves	Higher transpiration efficiency	Zheng et al., 2015
*ERECTA*	Arabidopsis	Arabidopsis mutation	Increase stomata density and reduced size, carbon isotope discrimination, photosynthesis	Masle et al., 2005
*GTL2-LIKE1* (*GTL1*)	Arabidopsis	Arabidopsis mutation	Reduced stomatal density and lowered transpiration without any effect on biomass	Yoo et al., 2010
*TaERF3*	Wheat	Overexpression in wheat	Drought and salinity tolerance	Rong et al., 2014
*TaERF1*	Wheat	Overexpression in Arabidopsis	Drought, salt, and low temperature tolerance	Xu et al., 2007
*AtERF019*	Arabidopsis	Overexpression in Arabidopsis	Drought tolerance, smaller stomata aperture, and lower transpiration rate	Scarpeci et al., 2017
*DREB1A*	Arabidopsis	Stress induced expression in wheat	Delayed water stress symptoms	Pellegrineschi et al., 2004
*TAZFP34*	Wheat	Overexpression in wheat roots	Increased root:shoot ratio	Chang et al., 2016;
*TaWRKY10*	Wheat	Overexpression in tobacco	Enhanced drought tolerance	Wang et al., 2013
*TaWRKY1* and *TaWRKY33*	Wheat	Overexpression in Arabidopsis	Enhanced drought and heat tolerance	He G.-H. et al., 2016
*TaWRKY1*	Wheat	Overexpression in tobacco	Enhanced drought tolerance and higher biomass under drought stress	Ding et al., 2016
*TaWRKY93*	Wheat	Overexpression in Arabidopsis	Enhanced drought, salt, and low temperature tolerance	Qin et al., 2015
*TaWRKY44*	Wheat	Overexpression in tobacco	Drought, salt, and osmotic stress tolerance	Wang F. et al., 2015
*RAP2.1*	Arabidopsis	Mutation in the gene	Enhanced drought and frost tolerance	Dong and Liu, 2010
*TaRAP2.1*	Wheat	Mutant overexpression in wheat	Drought tolerance	Amalraj et al., 2016
*SodERF3*	Sugercane	Overexpression in tobacco	Drought and osmotic tolerance	Trujillo et al., 2009
*OsERF4a*	Rice	Overexpression in rice	Enhanced drought tolerance	Joo et al., 2013

**Table 4:** Examples of EAR-motif containing transcriptional repressors involved in modulation of drought response.

**EAR repressor**	**Plant species**	**Sequence of the EAR motif**	**Function**	**References**
**ERF FAMILY**				
*AtERF7*	Arabidopsis	DLNFPP	AtERF7 binds to the GCC box and acts as a transcriptional repressor in ABA and drought stress responses.	Song et al., 2005
*OsERF3*	Rice	DLNRPP	EAR motif in OsERF3 is required to transcriptionally regulate ethylene biosynthesis and drought tolerance.	Zhang et al., 2013
*OsERF4a*	Rice	DLNLPP	OsERF4a improves drought tolerance through the repression of a downstream suppressor of stress response gene, Sir2.	Joo et al., 2013
*GmERF6*	Rice	DLNVPP	GmERF6 functions as an EAR repressor to downregulate expression of *AtKin1, AtPR3 and AtRD22* in transgenic *A. thaliana*.	Zhai et al., 2013
*SlERF36*	Tomato	DLNFPP	The EAR motif in SlERF36 controls early flowering and senescence and is responsible for reduction of stomatal density and photosynthesis.	Upadhyay et al., 2013, 2014
**DREB FAMILY**				
*TaRAP2.1L*	Wheat	DLNREP	EAR motif of TaRAP2.1L is responsible for a negative effect on wheat development and growth, and drought tolerance.	Amalraj et al., 2016
*RAP2.1*	Arabidopsis	DLNQIP	AtRAP2.1 negatively regulates DREB-type activators resulting in reduced tolerance to cold and drought stresses.	Dong and Liu, 2010
**C2H2 FAMILY**				
*ZAT7*	Arabidopsis	LDLDL	The EAR-motif of ZAT7 plays a key role in the defense responses of Arabidopsis to abiotic stresses.	Ciftci-Yilmaz et al., 2007
*ZAT10*	Arabidopsis	DLNIP	ZAT10 plays a key role of positive and negative regulator of plant defenses.	Mittler et al., 2006
*ZFP36*	Rice	DLNLMP	ZFP36 is required for ABA-induced antioxidant defense. The role of EAR motif in ZFP36 is yet to be determined.	Zhang et al., 2014
*AZF1 and AZF2*	Arabidopsis	LDLNL	AZF1 and AZF2 negatively regulate abscisic acid-repressive and auxin-inducible genes under abiotic stress conditions.	Kodaira et al., 2011
**MYB FAMILY**				
*MYB44*	Arabidopsis	LSLSL	Although the role of EAR motif is not defined, dominant repression by MYB44 causes oxidative damage and hypersensitivity to abiotic stresses.	Persak and Pitzschke, 2014

## Enhanced root growth for drought tolerance

Root system architecture (RSA) has been the target of wheat research and breeding to develop drought tolerant cultivars. Through QTL analysis, *DRO1* was identified as a regulator of RSA by modulating root growth angle in rice (Uga et al., 2011, 2013). Kinandang Patong (a *japonica* upland rice) containing a full-length *DRO1* copy exhibits deeper RSA; whereas, IR64 (an *indica* lowland rice) carrying a truncated copy, due to an insertion of 1 bp deletion within exon 4 resulting in introduction of a pre-mature stop codon of *DRO1*, exhibits shallower roots (Uga et al., 2013). The deep rooting was found to be beneficial in rice for not only drought tolerance but higher harvest index, nitrogen uptake, and flux of cytokinin from root to shoot during grain filling (Arai-Sanoh et al., 2014). These findings confirmed the positive contribution of root depth to drought avoidance facilitated by the ability to access moisture from deeper soil layers, better photosynthesis, and grain filling under drought conditions.

*DRO1* orthologs are found in many different plants, including both dicot and monocot species (Guseman et al., 2017). Consistent with the findings in rice, orthologs of *DRO1* in Arabidopsis and *Prunus* species were also found to influence the RSA, as evident from deeper rooting phenotypes in Arabidopsis and *Prunus* lines overexpressing *DRO1* (Guseman et al., 2017). The wheat genome harbors three copies of *DRO1* orthologs (TRIAE_CS42_5AL_TGACv1_374418_AA1199770, TRIAE_CS42_5BL_TGACv1_405332_AA1325250, and TRIAE_CS42_5DL_TGACv1_433409_AA1412320).

Notably, the wheat *DRO1* orthologs share 76% identitity with rice *DRO1*, suggesting the likelihood of functional similarity and potential applications in altering RSA for drought avoidance in wheat.

Harmonal regulation of root development in wheat is another interesting area that has attracted attension of wheat researchers. The recent functional characterization of ASYMMETRIC LEAVES2/LATERAL ORGAN BOUNDARIES DOMAIN (AS2/LBD) genes in wheat identified a transcription factor involved in root architecture enhancement. MORE ROOT from the D-genome of wheat (TaMOR-D), an auxin responsive transcription factor in the LBD family, when over-expressed in rice and Arabidopsis resulted in lateral root enhancement in Arabidopsis, and more crown roots, longer panicles and higher grain yield in rice (Li et al., 2016). It will be interesting to assess the response of these overexpression lines from Arabidopsis and rice to drought stress. As deeper and more effective root systems improve the capture of water from soil, further characterization of *DRO1* orthologs and *TaMOR* genes would provide an effective strategy for improvement of drought tolerance.

## Stomatal traits for enhanced drought tolerance

Stomata is the above ground control point for the entry of carbon dioxide (CO_2_) for photosynthesis and exit of water from plants via transpiration (Shahinnia et al., 2016). Stomatal closure as a response to stress leads to decreased leaf water potential, reduced carbon assimilation, oxidative stress, and increased canopy temperature (Ludlow and Muchow, 1990; Yokota et al., 2002). Maintaining better stomatal control over transpiration is critical for combating photosynthesis inhibition under drought stress (Bota et al., 2004). Stomatal pore area per leaf is determined by stomatal density and stomatal size. Significant genetic variation for stomatal size and density has been reported in wheat (Baloch et al., 2013; Shahinnia et al., 2016). Molecular understanding of genes regulating stomatal patterning and size in wheat is very important as this knowledge could be successfully employed to improve TE under drought stress. Smaller stomata size and higher density in wheat flag leaves were found to be associated with drought tolerance in wheat varieties (Baloch et al., 2013; Shahinnia et al., 2016). At least 40 genes in Arabidopsis are known to regulate stomatal development and patterning (Pillitteri and Torii, 2012). Interestingly, larger stomata and smaller density leads to better TE in Arabidopsis (Masle et al., 2005) but smaller stomata and higher density promotes higher TE in wheat (Baloch et al., 2013; Shahinnia et al., 2016). Molecular genetic understanding of genes and networks for stomatal patterning, size, and density regulation in wheat will enable modulation of stomatal index in wheat and improve TE under drought stress.

Transpiration efficiency is measured as biomass produced per unit of water transpired by a plant (Condon et al., 2004). Transpiration efficiency is connected to deeper root system and hence these two traits need to be simultaneously improved. Crop varieties that extract moisture from deeper zones (60–120 cm), maintain higher stomatal conductance and are able to maintain cooler canopy temperature (Pask and Reynolds, 2013). The stacking of deep root biomass and TE traits in wheat varieties will enhance protection from drought stress.

One of the classical examples of genes regulating TE is the *ERECTA* from Arabidopsis (Masle et al., 2005), a putative leucine-rich repeat receptor-like kinase (LRR-RLK) known to regulate stomatal density, epidermal cell expansion and patterning, mesophyll cell proliferation and cell-to-cell contact. Mutation of *ERECTA* in Arabidopsis increased stomatal density (resulting in increased stomatal conductance), decreased epidermal cell size and also decreased TE (by around 20%). Interestingly, *ERECTA* gene mutation can increase stomatal density without changing stomatal index. *ERECTA* thus regulates both photosynthesis ability as well as stomatal control over TE (Masle et al., 2005).

Two homologs of *ERECTA* in the wheat genome *TaER1* and *TaER2* were recently characterized (Zheng et al., 2015). Analysis of the expression patterns of *TaER1* and *TaER2* revealed a stronger negative correlation with carbon isotope discrimination, stomatal density, and transpiration rate but a positive correlation with flag leaf area, instant water use efficiency, biomass, and yield per plant, suggesting TaERs are involved in TE related traits and yield in bread wheat (Zheng et al., 2015). Interestingly, QTLs for stomatal density have been mapped to chromosome 7A on which one of the *TaER2* homoeologues resides (Huang et al., 2013; Zheng et al., 2015; Shahinnia et al., 2016). Further characterization of allelic diversity in TaER genes is essential prior to their future deployment in improving both drought tolerance and agronomic traits in wheat.

Stomata are key regulators of internal plant water status and carbon assimilation. Stomatal pores control both uptake of CO_2_ and water use through controlling transpiration rate, thus has a major role in photosynthesis as well as TE. Arabidopsis mutants with reduced stomatal density and increased stomata size showed better drought tolerance through reduced transpiration and higher biomass accumulation under drought stress (Pillitteri and Torii, 2012). Other environmental factors such as, temperature, water availability, and humidity also could be important modulators of photosynthesis. In contrast to *ERECTA* gene in Arabidopsis, *GTL2-LIKE1* (*GTL1*) controls stomata density, transpiration, and water use efficiency by repressing *STOMATAL DENSITY AND DISTRIBUTION 1* (*SDD1*) gene (Yoo et al., 2010). *GTL1* mutation reduced stomatal density and lowered transpiration without negative impact on CO_2_ assimilation and biomass production (Yoo et al., 2010) to improve photosynthesis efficiency in Arabidopsis.

Stomata pores cover only around 5% of the leaf area but contribute to around 70% water loss by plants (Hetherington and Woodward, 2003). Both stomatal conductance and stomatal index have an influence on carbon isotope discrimination (Δ^13^C). For example, Australian wheat varieties Drysdale and Rees developed for low Δ^13^C exhibit higher TE and around 10% better yield under dry and hot climate (Passioura, 2006; Richards, 2006). In environments where crops were able to maintain better water status, the genotypes exhibit a positive relationship between Δ^13^C and grain yields (Araus et al., 1998). Stomatal density and size in the complex wheat genome are dependent on ploidy level. Khazaei et al. (2010) compared *Triticum monococcum* (diploid), *T. durum* (tetraploid), and *T. aestivum* (hexaploid) for stomatal density and size, and observed a significant genetic variation for stomatal density. The high heritability of this trait enables dissection at molecular level (Bhagwat and Bhatia, 1993). Recently, Shahinnia et al. (2016) mapped QTLs for stomatal traits (size and density) on chromosomes 1A, 1B, 2B, and 7A in wheat. Interestingly, the 7A QTL co-localized with other QTLs for yield contributing traits such as, seeds per head, harvest index, and yield. Flag leaf may contribute up to 30–50% of the assimilates during grain filling (Sylvester-Bradley et al., 1990), hence flag leaf stomatal features are important to cope drought episodes.

**Figure 1:**
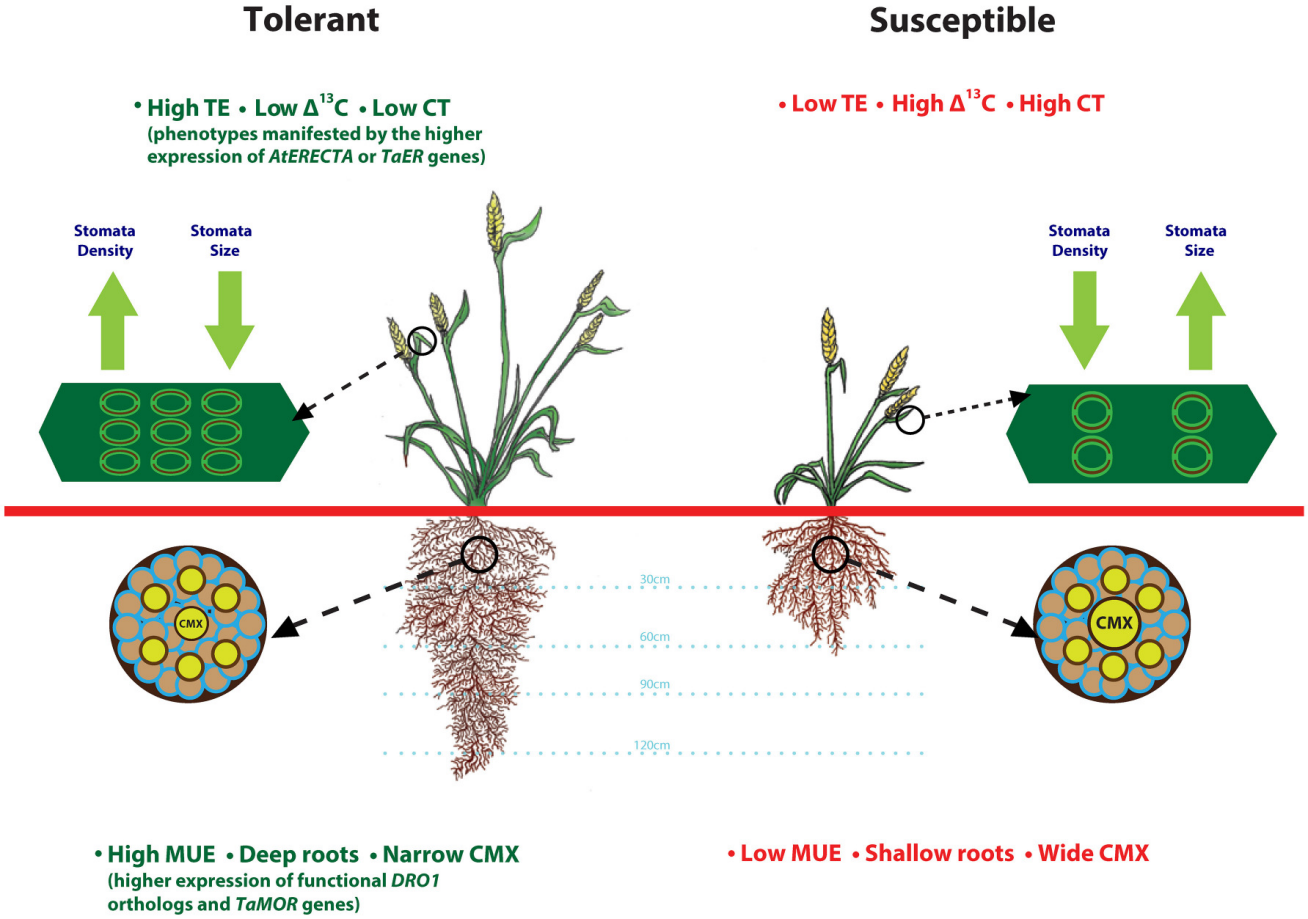
Root and stomatal traits that define drought tolerant and susceptible wheat plant ideotypes. This illustration is prepared based on the findings from various articles cited throughout the manuscript. Plant tolerance to drought stress relies on favorable root anatomical features such as, deeper roots and smaller central metaxylem (CMX) that contribute to improved moisture uptake-efficiency (MUE), and stomatal features such as high density and smaller size that contribute to lower canopy temperature (CT) and reduced carbon isotope discrimination.

Simultaneous improvements to root architecutre and depth for enhanced access to water from deeper soil layers and TE to preserve above ground water would be a valuable strategy that can be adopted to improve drought tolerance. A heat and drought tolerant wheat variety RAC875 had smaller and numerous stomata on flag leaf as compared to drought susceptible cultivar Kukri (Shahinnia et al., 2016). Root anatomical features such as, smaller central metaxylem (CMX) vessels and stele area complement RAC875 variety stomatal features by restricting root hydraulic conductivity under stress, another possible mechanism to reduce transpirational loss (Schoppach et al., 2014). Deeper roots enhance moisture uptake (Uga et al., 2013), whereas transcription factors from LBD gene family improve root architecture (Li et al., 2016). Drought tolerant spring wheat cultivars Anmol, Moomal, Bhittai, Sarsabz have smaller stomata size, lower stomatal conductance and are able to maintain higher relative water content under drought stress (Baloch et al., 2013). Based on these observations, we present a drought-tolerant wheat ideotype (Figure 1) with integration of adaptive root and stomatal traits including, deeper roots, narrower CMX, high moisture use and transpiration efficiency, and lower carbon discrimination and canopy temperature. Future research in wheat focused on understanding of genetic regulation of these complex traits would enable improvement of TE and MUE simultaneously.

## Regulation of gene expression under drought stress

Plants show remarkable transcriptional plasticity that allows them to thrive under harsh environmental conditions. Regulation of gene expression occurring at both the transcriptional and post-transcriptional level has a central role in plant's adaptation to environmental changes (Lopez-Maury et al., 2008). Gene regulation at the transcriptional level is coordinated by a complex network of functionally diverse regulatory proteins, including activators, repressors, co-activators, co-repressors, and chromatin modifiers. Positive regulation of gene expression in plants in response to drought stress has been well studied and extensively reviewed (Gahlaut et al., 2016; Joshi et al., 2016). Here, we highlight a few interesting discoveries of transcription factors that positively regulate adaptation responses to drought stress in wheat (summarized in Table 3).

### Positive regulation of gene expression under drought stress

AP2/ERF family transcription factors are well documented in many crop species for their role as mediators of both abiotic and biotic stress responses in plants (Licausi et al., 2013). AP2/ERF transcription factors are classified into four sub-families in wheat, including DREB, ERF, AP2, and RAV (Zhuang et al., 2011). ERFs are rapidly upregulated in response to stresses (He et al., 2011), and have been the subject of various overexpression studies to assess their usefulness in improving drought tolerance. Overexpression of *TaERF3* in wheat resulted in enhancement of drought as well as salinity tolerance (Rong et al., 2014), potentially due to the increase in accumulation of proline as well as chlorophyll content as compared to non-transformed lines and activation of a number of downstream genes by binding to the GCC-box *cis-*elements present in the promoter regions of target genes (Rong et al., 2014). Overexpression of another wheat ERF, *TaERF1*, activated stress-related genes, including *PR* and *COR/RD* genes, under normal growth conditions, and improved drought, cold, and salt tolerance in transgenic plants (Xu et al., 2007). A recent study of *AtERF019* indicated its role in drought tolerance with a phenotype of delayed flowering and maturity under drought stress suggesting overexpression of its orthologs could be used for obtaining enhanced drought tolerance in wheat without any compensation of the seed set (Scarpeci et al., 2017). ABA and ethylene are important phytohormones related to stress response regulation in plants. Induction of *TaERF3* by ethylene (Zhang et al., 2007) and ABA (Rong et al., 2014), and the presence of GCC boxes (ethylene response elements) as well as ACGT and ABRE (response elements for ABA) in the promoter confirms well established complex hormone-gene interplay mechanisms in abiotic stress tolerance. As ERFs are involved in multiple stresses, it would be useful to identify the conserved domains/motifs in their sequences and analyze their correspondence to different stress tolerance mechanisms. DREB and HSFs are key regulators of complex heat-drought stress genetic networks in wheat. Stress induced expression of *DREB1A* from Arabidopsis enhanced drought tolerance in wheat, indicating a promising role for DREBs in improving wheat adaptation to drought stress (Pellegrineschi et al., 2004).

Zinc Finger Proteins (ZFPs) with a QALGGH conserved domain are known to be associated with modulation of gene expression under drought stress (Cheuk and Houde, 2016). Role of ZFPs in drought tolerance in wheat (Cheuk and Houde, 2016), rice (Zhang et al., 2014), and Arabidopsis (Hichri et al., 2014) has been well established. A number of ZFPs, such as *ZFP182* and *ZFP252* (rice), *ZZ1* (soybean), *ZFP1* (Glycine soja) and *AZF1, AZF2, ZAT10*, and *ZAT11* (Arabidopsis) are known to be having a positive role in drought stress tolerance (Huang et al., 2012; Luo et al., 2012). *TaZFP22, TaZFP34*, and *TaZFP46* are root expressed and drought induced Q-type C2H2 zinc finger transcriptional repressors in wheat (Chang et al., 2016). *TaZFP34* was found to be up-regulated in response to multiple abiotic stresses, including heat, dehydration, salinity, and cold. Overexpressing this gene in wheat roots enhanced root:shoot ratio by reducing shoot growth while maintaining root elongation (Chang et al., 2016).

WRKY transcription factors are also important in critical plant developmental and physiological functions (Phukan et al., 2016). This gene family has been extensively studied for its role in abiotic stress tolerance in many plant species, including wheat (Ding et al., 2016), soybean (Wang F. et al., 2015), canola (He Y. et al., 2016), and common bean (Wu et al., 2017). A direct role of WRKY transcription factors in drought tolerance is evident from their up-regulation at the protein level in response to drought stress (Tripathi et al., 2014). Wheat WRKY transcription factors, *TaWRKY44* and *TaWRKY93*, were identified to be critical response factors under drought stress (Qin et al., 2015; Wang X. et al., 2015). A functional proof of the role of WRKY in drought tolerance of wheat was evident from overexpression of *TaWRKY10* in tobacco which enhanced drought tolerance response in transgenic tobacco lines with a suggested role as a negative regulator of antioxidant accumulation (Wang et al., 2013). Some of the wheat *TaWRKYs* (*TaWRKY*16, 24, 59, 61, and 82) were found to be differentially expressed in both leaf and root tissues under drought stress (Okay et al., 2014). A recent study identified *TaWRKY1* and *TaWRKY33* as candidates putatively involved in drought tolerance (He G.-H. et al., 2016); these genes when overexpressed in Arabidopsis lead to enhanced drought and heat tolerance. A positive role of *TaWRKY93* in drought, heat, salt tolerance as well as root growth enhancement was also evident from enhanced cellular membrane stability in Arabidopsis overexpression lines (Qin et al., 2015). Similarly, overexpression of an ABA dependent *TaWRKY1* in tobacco enhanced drought tolerance *via* stomatal closure to reduce water loss and altered osmotic adjustment to accumulate higher biomass (Ding et al., 2016). The role of *TaWRKY44* in drought and salinity tolerance *via* activating reactive oxygen species or antioxidant pathways from overexpression studies in tobacco provides a promising example of multiple roles of transcription factors and their possible use in developing wheat lines with tolerance to drought, salt, and osmotic stresses (Wang X. et al., 2015). Above studies also indicate the importance of studying genome wide variation in various transcription factors and exploitation of the allelic variation to enhance drought tolerance.

### Negative regulation of gene expression under drought stress

Although the positive control of transcriptional activation has been well studied, until recently relatively little was known about the negative regulation of gene expression in response to environmental changes. In the past decade, notable progress has been made in elucidating the molecular nature and functions of transcriptional repressors (Payankaulam et al., 2010). A few active transcriptional repression motifs, including EAR (ethylene-responsive element binding factor-associated amphiphilic repression motif; Ohta et al., 2001), TLLLFR (Matsui et al., 2008), R/KLFGV (Ikeda and Ohme-Takagi, 2009), and LxLxPP (Paponov et al., 2009), have been identified to facilitate recruitment of transcriptional co-repressors and chromatin modifiers to abate gene expression (Kagale and Rozwadowski, 2011). The EAR motif is the most pre-dominant form of active transcriptional repression motif identified in plants so far and is highly conserved across evolutionarily diverse plant species (Kagale and Rozwadowski, 2010, 2011; Kagale et al., 2010; Sherif et al., 2013; Upadhyay et al., 2014; Dong et al., 2015; Amalraj et al., 2016; Ma et al., 2017). Here, we will focus on EAR motif containing transcriptional repressors and their potential modes of action.

The EAR motif, defined by the consensus sequence patterns LxLxL or DLNxxP, is found in numerous transcriptional repressors in plants that negatively regulate genes involved in developmental, hormonal, and stress signaling pathways (Kagale et al., 2010). It was initially identified in a subset of class-II ERFs and TFIIIA-type ZFPs (Ohta et al., 2001). Interestingly, when this motif was tethered to transcriptional activators, they functioned as dominant repressors (Hiratsu et al., 2003). The Arabidopsis EAR repressome comprises 219 transcriptional regulators belonging to 21 different families (Kagale et al., 2010). EAR repressors suppress the expression of target genes through chromatin modification *via* physically interacting with co-repressors such as, *SAP18*, known to directly interact with a histone deacetylase (*HDA19*) potentially forming a repression complex, or *TOPLESS* (*TPL*) which is also known to function in conjunction with *HDA19* (Szemenyei et al., 2008; Pauwels et al., 2010; Kagale and Rozwadowski, 2011; Oh et al., 2014; Ma et al., 2017).

EAR-mediated transcriptional repression has emerged as one of the principal mechanisms for negative regulation of gene expression in response to abiotic stresses in plants. A number of EAR-motif containing transcriptional repressors from various plant species have been functionally characterized and their role in drought stress responses has been established (Table 4). The Arabidopsis gene *RAP2.1*, a DREB gene with an EAR motif, is strongly induced by drought and cold stresses (Dong and Liu, 2010). Overexpression of *RAP2.1* leads to enhanced sensitivity to drought and cold stresses; whereas, T-DNA insertion mutant alleles of this gene exhibit enhanced tolerance to drought. Stress-induced expression of *RD/COR* genes was repressed in *RAP2.1* overexpressing lines but increased in mutant lines (Dong and Liu, 2010). Similarly, overexpression of wheat ortholog of *RAP2.1* (*TaRAP2.1*) led to dwarfism and frost sensitivity; whereas overexpression of its EAR-motif inactivated variant enhanced its ability to survive drought and frost (Amalraj et al., 2016). Overexpression of several other EAR motif-containing ERF/DREB proteins also results in decreased drought tolerance *via* reduced expression of stress defense genes (Song et al., 2005; Pan et al., 2010; Zhang et al., 2013; Upadhyay et al., 2014; Amalraj et al., 2016; Scarpeci et al., 2017). The complete deletion or mutation of conserved residues within the EAR motif in various transcriptional repressors has been shown to alter or even reverse their functions (Dong and Liu, 2010; Pan et al., 2010; Zhang et al., 2013; Amalraj et al., 2016). Remarkably, overexpression of an ERF protein from sugarcane (*SodERF3*) in tobacco increased tolerance to drought and osmotic stresses (Trujillo et al., 2009). The EAR motif in *SodERF3* is different from other ERF proteins, such that the highly conserved proline residue in the DLNxxP type of EAR motifs found in various ERFs is replaced by leucine in *SodERF3* (Trujillo et al., 2009). It may be speculated that this proline to leucine change in *SodERF3* renders its EAR-motif inactive thereby converting it into a transcriptional activator.

Sustained activation of stress responses is metabolically expensive, and therefore plants have evolved negative gene regulation mechanisms to keep it under tight control during normal growth and development and also during stress conditions (Kazan, 2006). It is speculated that the EAR-motif containing ERF repressors perform “capping” role by limiting the upper levels of stress-responsive genes and thereby exerting strong regulatory control over stress responses (Dong and Liu, 2010; Amalraj et al., 2016).

Interestingly, a few EAR-motif containing transcriptional regulators are shown to function as positive regulators of drought responses in plants. For example, *OsERF4a* (an EAR motif containing ERF) when overexpressed in rice results in increased drought tolerance (Joo et al., 2013). Intriguingly, *DRO1* in rice and its orthologs in other plant species also contain a conserved motif (IVLEI) in the C-terminal region, which is required for *DRO1* overexpression phenotypes of deeper root in *Arabdiopsis* and *Prunus* species (Guseman et al., 2017) and is very similar to the EAR motif. It is possible that the EAR-motifs in the positive regulators enhance stress tolerance by suppressing the expression of other negative regulators of stress responses. Indeed, *OsERF4a* is shown to improve drought tolerance through the repression of another downstream suppressor of stress response gene, *Sir2* (Joo et al., 2013). In summary, EAR-motif containing repressors despite being negative and/or positive regulators of gene expression play a key role in modulating plant stress responses.

Notably, the past efforts to characterize both positive and negative regulators of drought tolerance in wheat and other species have involved their genetic manipulation by transgenic approaches. The commercialization of transgenic wheat has been problematic due to regulatory hurdles in Europe and Africa. Thus, the future deployment of transcriptional regulators in wheat breeding programs should involve screening of natural allelic variation through EcoTilling approaches or generation of variation through non-transgenic gene editing approaches. The availability of a new gold-standard genome sequence of wheat produced by the International Wheat Genome Sequencing Consortium (IWGSC) and the recent advances made in genome editing technologies provide valuable resources and technologies for non-transgenic manipulation of the wheat genome to improve drought-related traits.

## Concluding remarks

Crop growth and development are greatly affected by changes in morphological and physiological responses resulting from the lack of soil moisture. So far only a small number of genes, such as, *TaMOR, TaER*s, and a few transcriptional regulators, affecting drought-adaptive traits in wheat have been fully investigated. Comparatively, the drought tolerance mechanisms have been extensively studied in other model species and crop plants, including Arabidopsis, rice, and maize. Functional validation of the wheat orthologs of drought tolerance genes identified in other species is essential for their deployment in breeding drought tolerant cultivars.

Combining deep roots capability with superior anatomical features, such as, smaller xylem diameter and increased stomatal density helps wheat to extract and use water to maintain normal photosynthesis under depleting soil moisture conditions. In addition, a tightly linked interaction between xylem-stomatal physiologies is a must to cope with drought episodes. The comprehensive gene expression analyses combined with both forward and reverse genetic approaches have identified several target genes that are useful in manipulating an acclimation response to drought stress in plants. Future elucidation of the precise mode of action of several uncharacterized genes by analyzing phenotypic effects of their mutant variants would significantly advance our understanding of the regulatory networks underlying the complex drought tolerance mechanisms and enable their utilization in developing drought tolerant cultivars.

Recent sequencing of the wheat genome has opened up tremendous opportunities to understand the complex architecture of drought tolerance mechanisms. Wheat harbors a very large and complex allohexaploid genome of 17 Gb with ~80% of repetitive elements and an estimated 124,201 annotated genes (International Wheat Genome Sequencing Consortium, 2014). Similar to other neopolyploid species, such as, brassicas, cotton, and maize, homoeolog expression bias which refers to the preferential or altered expression of one homeolog over the other is observed in wheat as about 55% of the wheat genes were reported to be expressed only from one or two homoeologs of the genome. Transcriptomic or microarray based gene expression analysis has been used as an important tool to understand transcript modulation under stress. The wheat genome sequence combined with transcriptome, proteome, and metabolome profiling of genes associated with various traits for drought tolerance will aid in overcoming the challenges posed by genome complexity and facilitate the analysis of genetic basis of drought tolerance in wheat. Furthermore, it will help in integration of phenotypic, biochemical, and genomics-assisted selection strategies for breeding drought tolerant wheat cultivars.

## Author contributions

MK, RS, and SK discussed and prepared the outline. MK did the literature search and prepared the first draft. RS and SK revised, edited and contributed to writing the manuscript. SO, YU, and MS contributed to discussions and critical revision of the manuscript.

### Conflict of interest statement

The authors declare that the research was conducted in the absence of any commercial or financial relationships that could be construed as a potential conflict of interest. The handling Editor declared a shared affiliation, though no other collaboration, with several of the authors MK and SK.
